# Prevalence of tuberculosis/COVID-19 co-infection and factors associated with SARS-CoV-2 infection in pulmonary tuberculosis patients at a respiratory diseases center: a cross-sectional study

**DOI:** 10.11604/pamj.2023.44.204.38541

**Published:** 2023-04-28

**Authors:** Laurent-Mireille Endale Mangamba, Christiane Ingrid Medi Sike, Joël Noutakdie Tochie, Grace Ngondi Dalle, Nadia Nkouagmi, Adamou Dodo Balkissou, Bernard Eyoum Bille, Bertrand Hugo Mbatchou Ngahane, Carole Else Eboumbou Moukoko

**Affiliations:** 1 Faculty of Health Sciences, University of Buea, Buea, Cameroon,; 2 Douala Laquintinie Hospital, Douala, Cameroon,; 3 Faculty of Medicine and Biomedical Sciences, University of Yaoundé I, Yaoundé, Cameroon,; 4 Faculty of Medicine and Pharmaceutical Sciences, University of Douala, Douala, Cameroon,; 5 Faculty of Medicine and Biomedical Sciences of Garoua, University of N´Gaoundéré, Garoua, Cameroon,; 6 Douala General Hospital, Douala, Cameroon; 7 Malaria Unit, Centre Pasteur Cameroon, Yaoundé, Cameroon

**Keywords:** COVID-19, tuberculosis, SARS-CoV-2, prevalence, carriage

## Abstract

**Introduction:**

currently, tuberculosis (TB) is the second cause of infectious disease-related deaths before COVID-19. These two infections have several similarities but little data is available on TB/COVID-19 co-infection, hence, we sought to investigate the prevalence of this co-infection and the factors associated with Severe Acute Respiratory Syndrome Coronavirus 2 (SARS-CoV-2) infection in tuberculosis patients in a tuberculosis-endemic area.

**Methods:**

we conducted a prospective cross-sectional study from January to June 2022 at Respiratory Diseases Center in Douala, Cameroon by enrolling all consenting pulmonary tuberculosis patients. The presence of SARS-CoV-2 ribonucleic acid (RNA) and gamma-interferon levels were laboratory analyzed using the Reverse Transcriptase-Polymerase Chain Reaction and the enzyme-linked immunosorbent assay (ELISA) technique, respectively. The factors associated with COVID-19 carriage in pulmonary tuberculosis patients were analyzed by logistic regressions.

**Results:**

overall, we enrolled 185 patients; 57.8% were males (sex ratio of 1.36) and their mean age was 43.70 ± 17.89 years. The prevalence of SARS-CoV-2 RNA in pulmonary TB patients was 24.3%. Asthma and sore throat were the factors favoring carriage (OR=3.74; 95% CI=1.271-11.017; p=0.017 and OR=4.05; 95%CI=1.204-13.600; p=0.024) and cough was a protective factor (OR=0.15; 95% CI = 0.034-0.690; p=0.015).

**Conclusion:**

the prevalence of SARS-CoV-2 carriage in tuberculosis patients is high and greater than the national prevalence. Asthma and sore throat would be associated factors.

## Introduction

Tuberculosis (TB) is a public health problem on a global scale. According to estimates by the World Health Organization (WHO), the global TB incidence rate is approximately 10 million cases per annum [1]. It is caused by the mycobacteria of the tuberculosis complex and more frequently by the pathogenic agent Mycobacterium tuberculosis (Mtb) [2]. In addition, TB is the second cause of global deaths related to infectious diseases before COVID-19 [3]. COVID-19, which is caused by SARS-CoV-2, a respiratory-tropic virus, first out broke in December 2019 in Hubei province in China before its worldwide spread [4]. As of May 22, 2022, the number of COVID-19 cases worldwide was 523,044,671 with 6,248,739 deaths. During the same period, 3,931,534 cases and 100,952 deaths were reported in sub-Saharan Africa (SSA) [5].

SARS-CoV-2 infection and Mtb infection have many similarities. Indeed, they are both transmitted through the air and then preferentially attack the lungs; their clinical presentations overlap at various points such as fever, cough and dyspnea [6]. These two conditions have many risk factors including advanced age, gender, and comorbidities such as diabetes mellitus, immunosuppression and chronic respiratory diseases. According to some authors, risk factors such as age and comorbidities are determinants of mortality in people co-infected with COVID-19/TB and, co-infection could be associated with the worsening of the disease and an increase in fatality rate [7-10]. Other research works suggest common protective factors such as the Bacille Calmette-Guérin (BCG) vaccine, and the level of interferon-gamma (IFN- γ) [11,12].

In Cameroon, the National Tuberculosis Control Program (PNLT) reported 22,499 new TB cases in 2020 [13]. The first case of COVID-19 was identified and confirmed on March 6, 2020 [14]. By May 22, 2022, 119,780 cases and 1,927 deaths were reported in Cameroon. On the same date, the vaccination coverage rate was 4.58% [5]. Given the coexistence of these two conditions, the potentially aggravating nature of the co-infection, and the lack of published data on TB/COVID-19 co-infection, we thought to assess the prevalence of TB/COVID-19 co-infection and to determine the factors associated with this SARS-CoV-2 carriage in TB patients in a TB-endemic area like Cameroon.

## Methods

**Study design, setting, and population:** this was an analytical cross-sectional study carried out prospectively between January and June 2022 by enrolling all patients irrespective of age, presenting at the Respiratory Diseases Center (RDC) of Douala Laquintinie Hospital (DLH). Laboratory analysis of samples took place at the Retrovirology laboratories for the research of SARS-CoV-2 of DLH for the dosage of IFN-γ. DLH is a category 2 health facility in the Cameroon health system pyramid. The RDC is a major referral center for the management of respiratory diseases and phthisis in Cameroon and surrounding regions including the Central African sub-region. Each year more than 1000 cases of all clinical forms of TB are diagnosed, managed and followed up at the Respiratory Disease Center. All TB patients hospitalized or seen in an outpatient setting during the study period were enrolled through a convenience consecutive sampling method and invited to participate. Were excluded patients who had had a SARS-CoV-2 infection two days before presentation, those with a negative reverse-transcriptase polymerase chain reaction (RT-PCR) COVID-19 test result, and those in whom nasopharyngeal and blood samples could not be obtained.

### Study clinical and laboratory procedures

**Data collection and study variables:** the diagnosis of tuberculosis was made in compliance with the standards of the National Tuberculosis Control Program (PNLT) and WHO recommendations [15]. After obtaining informed consent from all patients, we performed an interview administration of questionnaires to patients. We retained the following study variables: (i) socio-demographic factors: age, sex, occupation; ii) history and potential risk factors: Bacillus Calmette-Guerin (BCG) vaccination status, facial mask and physical distancing practices, asthma, HIV/AIDS infection, hypertension, diabetes mellitus, tobacco and alcohol abuse; iii) clinical characteristics: cough, dyspnea, asthenia, sore throat, headache, chest pain, weight loss, excessive sweating, shortness of breath. These variables constituted the associated factors that were compared to the RNA carriage of SARS-CoV-2.

Laboratory search for SARS-CoV-2 RNA: the nasopharyngeal sample was taken from both nostrils after the introduction of the swab to the posterior face of the pharynx. Once the sample was taken, the swab was sent directly at (2-8°C) to the molecular biology laboratory for analysis. This sample was stored in a refrigerator (2-8°C) for 48 h, -20°C for one week or -80°C until extraction when the analysis was deferred. The RNAs were stored in a refrigerator at (2-8°C) for immediate amplification, at -20°C for amplification within 24 h or at -70°C for amplification over 24 h. The test was performed by PCR for SARS-CoV-2 using the DaAn protocol (Guangzhou, Guangdong, China). Total nucleic acid from nasopharyngeal swabs in the viral transport medium (VTM) was extracted using a DaAn Gene extraction kit (DaAn Gene Co, Ltd., Sun University Yat-sen, China®) according to the manufacturer's instructions for virus inactivation and RNA extraction. The DaAn Gene 2019-nCoV detection kit (DaAn Gene Co, Ltd., of Sun Yat-sen University, China®) was used for amplification. The amplification principle was based on a set of primers and probes designed to detect sequences of ORF1ab genes, SARS-CoV-2 nucleocapsid (N) protein, and the human ribonuclease P (RNP) gene developed as a target gene for the internal control of swab monitoring of samples, the extraction process and the amplification process. Probe detection modes were defined as follows; ORF1ab: VIC, Quencher: NONE. N-Gene: FAM, Quencher: NONE. - internal control: Cy5, Quencher: NONE. Passive reference: NONE. The amplification process took place on the Quan studio 7flex automaton (Strasbourg, France), the cycle threshold (Ct) was set based on laboratory verification and determination of the appropriate Ct for the target genes. The result is interpreted as positive or negative. Laboratory assay of gamma-interferon: a volume of 5ml of venous blood was collected in a dry tube. The serum was obtained after centrifugation of the dry tube at 1500 rates/minute. The supernatant was collected immediately tested or and stored at -20°C before the analysis.

The analysis was carried out by Enzyme-Linked Immuno-sorbent Assay (ELISA) technique using the IFN-γ-ELISA kit (DIA source Immuno-Assay, Louvain-la-Neuve, Belgium®). A volume of 50 µl of sample was used for the analyses. The test was performed on microplates, the principle was based on the use of monoclonal antibodies directed against distinct epitopes of IFN-γ. Calibrators and samples react with the monoclonal capture antibody coating the wells and with a monoclonal antibody labeled with peroxidase. After an incubation period, the microplate is washed to remove free enzymatically labeled antibody. A chromogenic solution is added and incubated. The reaction is stopped with the addition of Stop Solution and the microplate is then read at the appropriate wavelength of 450nm. The amount of substrate replacement is determined colorimetrically by measuring absorbance, which is proportional to IFN-γ concentration. A calibration curve was drawn in f (IFN γ) and the concentration of IFN-γ in the samples was determined by interpolation of the calibration curve. The normal value was set according to the manufacturer's recommendations and the interpretation of IFN γ results: < 0.58 (μg/ml) = normal; > 0.58(μg/ml) = above normal.

**Definitions of operational terms:** tuberculosis (TB) patient: this was any patient, presenting a positive result for the search for tuberculosis by microscopy or by Polymerase Chain Reaction (PCR) or persistent signs suggestive of tuberculosis after 10 days of broad-spectrum antibiotic treatment without any other obvious cause and after the decision to start treatment by the pulmonologist. COVID-19/TB co-infected: this was any TB patient whose SARS-CoV-2 RT-PCR was positive. Non-co-infected TB patient: this was any tuberculosis patient who was tested negative by RT-PCR for SARS-CoV-2 carriage. COVID-19/TB coinfection: this was any subject with tuberculosis whose SARS-CoV-2 RT-PCR was positive.

**Statistical analysis:** statistical analysis was performed using SPSS software version 23. Generally, quantitative data were presented in the form of means or medians, and standard deviations, whereas qualitative data were presented in the form of frequencies and numbers. Socio-demographic, relevant past histories/comorbidities were compared between COVID-19/TB co-infected patients and patients with TB only using the Chi-square test or Fisher exact test where appropriate. We performed a univariate analysis where the gross association between each variable and co-infection was studied. Variables with a p-value<0.15 were then included in the multivariate analysis for the assessment of independence on the development of COVID-19 infection (dichotomized as a yes/no variable) in TB patients. Adjusted odds ratios (OR), their corresponding 95% confidence intervals (95% CI), and p-values were reported. The level of statistical significance was set at a p-value < 0.05 in this study.

**Ethical considerations:** ethical clearance was obtained from the Institutional Review Board of the Faculty of Medicine and Pharmaceutical Sciences, University of Douala, Cameroon (N° 3061CEI-UDo/04/2022/M) and administrative authorization from the Directorate of DLH (N° 0980/AR/MINSANTE/DHL). This study was carried out in compliance with bioethical laws, the data protection act, as well as in accordance with good clinical practice and The Declaration of Helsinki. The teams and supervisors participating in the study were informed of the terms of the study at the start and at the end of the trial; the patient was informed of the study and at the end had signed the consent form.

## Results

**Figure 1:**
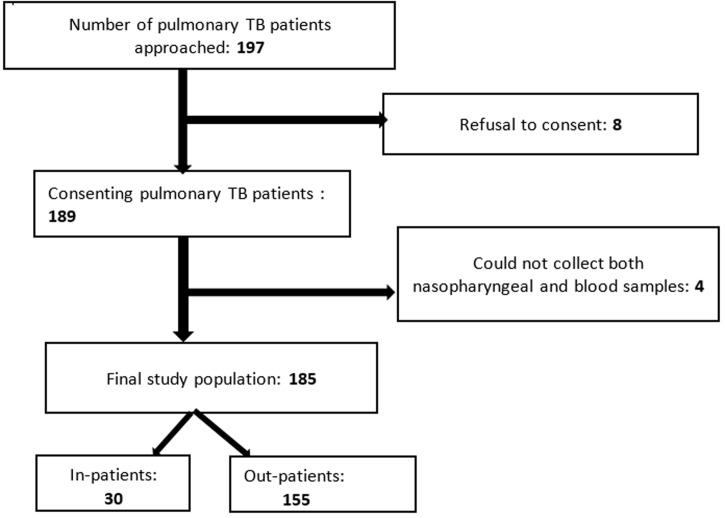
flow chart explaining the mode of selection

The flow diagram (Figure 1) explains how to select patients with pulmonary TB. Of 197 patients, 185 were included. Eight refused to participate and four patients who received a single type of sample (nasopharyngeal or blood swab) were excluded from the study giving a response rate of 93.9%.

**Socio-demographic factors:** Table 1 shows the baseline socio-demographic factors of TB patients. Their mean age was 43.70 ± 17.89 years, and the most represented age group was 45 to 55 years old, with a male predominance (53.3%), yielding a sex ratio of 1.36. The average gamma-interferon level was 0.18 ± 0.12. This gamma-interferon level was normal in 155 (83.8%) participants.

**Table 1:** socio-demographic factors of the study population

	Frequency (%)	N = 185			
Socio-demographic factors		COVID-19/ TB patients N = 45 n (%)	TB patients only N=140 n (%)	P-Value	OR (95% CI]
**Age ranges (in years)**					
(5-15)	8 (4.3)	3 (6.7)	5 (3.6)	0.830	1.20 (0.22 – 6.33)
(15-25)	24 (13)	8 (17.8)	16 (11.4)	Reference	
(25-35)	29 (15.7)	9 (20)	20 (14.3)	0.858	0.90 (0.28 - 2.86)
(35-45)	37 (20)	11 (24.4)	26 (18.57)	0.767	0.85 (0.28 - 2.55)
(45-55)	38 (20.5)	8 (17.8)	30 (21.4)	0.285	0.53 (0.16 -1.68)
(55-65)	21 (11.4)	3 (6.7)	18 (12.9)	0.148	0.33 (0.07 - 1.47)
≥ 65	28 (15.1)	3 (6.7)	25 (17.9)	0.057	0.24 (0.05 - 1.04)
**Gender**					
Male	107 (57.8)	24 (53.3)	83 (59.3)	0.482	1.27 (0.65 - 2.50)
Female	78 (42.2)	21 (46.7)	57 (40.7)		
**IFNγ (µg /ml)**					
Normal	155 (83.8)	35 (77.8)	120 (85.7)	0.169	1.82 (0.77 - 4.27)
Normal height	30 (16.2)	10 (22.2)	(14.3)		

IFN-γ: interferon-gamma; normal value: < 0.58 µg/ml, above normal value: > 0.58 µg/ml; TB: tuberculosis; CI: 95% confidence interval; p-value significant: < 0.05.

Overall, there were 45 (24.3%) TB patients tested positive for COVID-19 whose mean age was 43.59 ± 17.92 years with a sex ratio of 1.14. The most represented age group was from 45 - 55 years. The interferon gamma level was 0.19 ± 0.12 μg/ml. Among these co-infected patients, 35 (77.8%) had normal IFN-γ levels. There was no statistically significant difference in the distribution of socio-demographic factors among COVID-19/TB co-infected and non-co-infected-TB.

**Comorbidities of the study population:** Table 2 presents the past history of TB patients. HIV/AIDS was mainly represented in the population (52.9%) followed by hypertension (86.7%). BCG vaccine coverage was 90.7% and 15.7% had a history of TB. In co-infected patients, the most common comorbidities were HIV/AIDS (60%), hypertension (33.33%), kidney disease (28.9%) and diabetes mellitus (24.44%). Meanwhile, 86.7% of patients co-infected with COVID-19/TB had received BCG in childhood and 17.3% of patients had had TB at least once. There was an association between COVID-19/TB co-infection and asthma (OR: 3.56; 95% CI [1.25 - 10.15]; p=0.017).

**Table 2:** past medical history of the study population

	N = 185				
Past history	Frequency (%)	COVID-19/TB patients N = 45 n (%)	TB patients only N = 140 n (%)	p-value	OR [95% CI]
**Asthma**					
Yes	16 (8.6)	8 (17.8)	8 (5.7)	0.017	3.56 [1.25 – 10.15]
No	169 (91.4)	37 (82.2)	132 (94.3)		
**HIV/AIDS**					
Yes	101 (54.6)	27 (60)	74 (52.9)	0.403	1.33 [0.67 - 2.64]
No	84 (45.4)	18 (40)	66 (47.1)		
**Diabetes mellitus**					
Yes	34 (18.4)	11 (24.44)	23 (16.43)	0.230	1.64 [0.72 - 3.71]
No	151 (81.6)	34 (75.56)	117 (83.57)		
**Hypertension**					
Yes	74 (40)	15 (33.33)	59 (42.14)	0.295	1.45 [0.72-2.94]
No	111 (60)	30 (66.67)	81 (57.86)		
**Renal disease**					
Yes	54 (29.2)	13 (28.9)	41 (29.3)	0.959	1.01 [0.48-2.13]
No	131 (70.8)	32 (71.1)	99 (70.7)		
**Tobacco abuse**					
Yes	27 (14.6)	5 (11.1)	22 (15.7)	0.449	1.49 [0.53 - 4.19]
No	158 (85.4)	40 (88.9)	118 (84.3)		
**Alcohol abuse**					
Yes	56 (30.3)	10 (22.2)	46 (32.9)	0.180	1.71 [0.78 - 3.76]
No	129 (69.7)	35 (77.8)	94 (67.1)		
**COVID-19 past history**					
Yes	3 (1.6)	1 (2.2)	2 (1.4)	0.716	1.56 [0.13 - 17.71]
No	182 (98.4)	44 (97.8)	138 (98.6)		
**BCG immunization status**					
Yes	166 (89.7)	39 (86.7)	127 (90.7)	0.439	1.50 [0.53 - 4.21]
No	19 (10.3)	6 (13.3)	13 (9.3)		
**Past history of tuberculosis**					
Yes	30 (16.2)	8 (17.8)	22 (15.7)	0.744	1.16 [0.47 - 2.82]
No	155 (3.8)	37 (82.2)	118 (84.3)		

BCG: Bacille Calmette-Guérin; HIV: human immunodeficiency virus; OR: odds ratio; CI: confidence interval at 95°C

**Clinical characteristics of the study population:** Table 3 shows the clinical characteristics of tuberculosis patients. Cough was the most common clinical sign (97.9%) followed by chest pain (48.6%) and asthenia (44.3%) in TB non-co-infected patients. The main clinical signs reported during COVID-19/TB co-infection were cough (88.9%), asthenia (57.8%) and chest pain (53.3%). There was a significant difference in the distribution of clinical signs in co-infected COVID-19/TB and non-co-infected TB patients concerning cough and sore throat (OR: 5.70; 95% CI [1.30 - 24, 92] p=0.021 and OR 3.43; 95% CI: [1.04 - 11.25]; p=0.041).

**Table 3:** clinical characteristics of the study population

	N = 185				
Clinical signs	Frequency (%)	Covid-19/TB patients N = 45 n (%)	TB patients only N = 140 n (%)	p-value	OR [95% CI]
**Cough**					
Yes	177 (95.7)	40 (88.9)	137 (97.9)	0.021	5.70 [1.30 – 24.92]
No	8 (4.3)	5 (11.1)	3 (2.1)		
**Sore throat**					
Yes	12 (6.5)	6 (13.3)	6 (4.3)	0.041	3.43 [1.04 – 11.25]
No	173 (93.5)	39 (86.7)	134 (95.7)		
**Dyspnea**					
Yes	58 (31.4)	17 (37.8)	41 (28.3)	0.287	1.46 [0.72 - 2.96]
No	127 (68.6)	28 (62.2)	99 (70.7)		
**Asthenia**					
Yes	88 (47.6)	26 (57.8)	62 (44.3)	0.117	1.72 [0.87 - 3.39]
No	97 (52.4)	19 (42.2)	78 (55.7)		
**Headache**					
Yes	5 (2.7)	1 (2.2)	4 (2.9)	0.820	1.29 [0.14 – 11.88]
No	180 (97.3)	44 (97.8)	136 (97.1)		
**Chest pain**					
Yes	92 (49.7)	24 (53.3)	68 (48.6)	0.579	1.21 [0.61 - 2.37]
No	93 (50.3)	21 (46.7)	72 (51.4)		
**Fever**					
Yes	35 (18.9)	8 (17.8)	27 (48.6)	0.822	1.10 [0.46 - 2.64]
No	150 (81.1)	37 (82.2)	113 (51.4)		
**Weight lost**					
Yes	26 (14.1)	7 (15.6)	19 (17.1)	0.739	1.17 [0.45 - 3.00]
No	159 (85.9)	38 (84.4)	121 (82.9)		
**Dyspnea**					
Yes	31 (16.8)	7 (15.6)	24 (17.1)	0.804	1.12 [0.44 - 2.81]
No	154 (83.2)	38 (84.4)	116 (82.9)		
**Hypersudation**					
Yes	15 (8.1)	5 (11.1)	10 (7.1)	0.400	1.62 [0.052 - 5.03]
No	170 (91.9)	40 (88.9)	130 (92.9)		

CI: 95% confidence interval; aOR: adjusted odds ratio; TB: tuberculosis; OR: odds ratio

**Multivariate analysis of factors associated with the carriage of SARS-CoV-2 by tuberculosis patients:** in multivariate analysis (Table 4), asthma, cough and sore throat were significantly associated with SARS-CoV-2 RNA carriage. Asthma and sore throat appeared to be aggravating factors for SARS-CoV-2 infection and cough was a protective factor for COVID-19 (OR=0.15; 95% CI=0.034-0.690; P=0.015).

**Table 4:** multivariate analysis of factors associated with SARS-CoV-2 carriage by tuberculosis patients

Associated factors	COVID-19/TB patients N = 45	TB patients only N = 140				
**Asthma**	**n (%)**	**n (%)**	**OR [95% CI]**	**p-value**	**aOR [95% CI]**	**p-value**
Yes	8 (17.8)	8 (5.7)	3.56 [1.25 – 10.15]	0.017	3.74 [1.27-11.01]	0.017
No	37 (82.2)	132 (94.3)				
**Cough**						
Yes	40 (88.9)	137 (97.9)	0.17 [0.04 – 0.76]	0.021	0.15 [0.03-0.69]	0.015
No	5 (11.1)	3 (2.1)				
**sore throat**						
Yes	6 (13.3)	6 (4.3)	3.43 [1.04 – 11.25]	0.041	4.05 [1.20-13.60]	0.024
No	39 (86.7)	134 (95.7)				

aOR: adjusted odds ratio; TB: tuberculosis; OR: odds ratio

## Discussion

Our work aimed to assess the prevalence of SARS-CoV-2 RNA carriage in TB patients and to determine the factors associated with this carriage in a tuberculosis-endemic area represented by a major tuberculosis referral center in Cameroon. One hundred and eighty-five pulmonary TB patients were included with a male predominance of 57.8% (107/185) and a sex ratio of 1.36; this gender disparity in the prevalence of tuberculosis is also found in most countries with a high prevalence of tuberculosis [15] and there is no consensus on the reasons that can explain it [12]. However, the high prevalence of certain recognized risk factors for tuberculosis in male subjects could explain this [16].

The mean age of the study population was 43.70 ± 17.89 with extremes of 5 to 88 years, the age group of (45-55) years was the most affected with 20.5% of cases (38/185). This data is similar to that of the literature and corroborate the assertion that tuberculosis is a disease of young adults. Balkissou *et al*. showed a predominance of tuberculosis in young adults with a median age of 34 (27-44) years in Cameroon [17]. Tékpa *et al*. in Central African Republic and Boushab *et al*. in Mauritania found the same predominance with a respective mean age values of 35, 69 ± 10, 65 and 41 ± 16 years and 35 ± 10, 65 years [2,18]. Singled adults were the most affected participants (65.4%). This finding is comparable to the observations made by Tékpa *et al*. who found 54.4% of singles [2]. Cases with a history of TB in our work were 16.2% (30/185), a value significantly higher than the 8.64% found by Tékpa *et al*. [2]. The prevalence of associated factors may justify the country's policy in terms of control; in fact, the prevalence of tuberculosis infection varies according to the country [19]. In our work, tuberculosis was associated with one or more comorbidities, mainly with HIV/AIDS infection, with a seroprevalence of 54.6%, in subjects with a male predominance (59/107). In 2012, in Douala, Cameroon, Boushab *et al*. found a comparable result (37.6%) in 383 pulmonary tuberculosis patients [18]. This result is close to that obtained by Balkissou *et al*. in 2016 who reported a male predominance with a 44% HIV seropositivity rate [17]. In contrast, differs from those of Tékpa *et al*. reported a higher prevalence of HIV/AIDS (73.36%) among women. This difference would be related to the predominance of women in the internal medicine wards of the Friendship Hospital in Bangui [2]. On admission, more than half of TB patients had at least one clinical sign. The most common were cough (95.7%), and chest pain (49.7%). Tékpa *et al*. described chronic cough (71.81%) and chest pain (57.73%) as the most frequent clinical signs in tuberculosis [2].

The prevalence of COVID-19/TB co-infection was 24.3% and was dominant in men. This proportion is higher than those of Mousquer *et al*. in South Africa (9.53%), Karla *et al*. in 2020 the Philippines (20%) and Tadolini *et al*. in 2020 Belgium (18.3%) [10,20,21]. The age group of (35-45) years was the most affected by COVID-19/TB co-infection. However, there was no association between COVID-19/TB co-infection and age. Stochino *et al*. in 2020 in Northern Italy as well as Gupta *et al*. 2020 in India obtained similar results with respective median ages of 39 (27-49) years and 36 (27-59.5) years [11,22]. Cytokines have an important function in host defense; Abdel-Hamed *et al*. in 2020 in Egypt, studied the role of gamma-interferon in co-infections and in particular in co-infections of SARS-CoV-2 with digestive parasitizes, high levels of gamma-interferon (50, 13pg/ml) have been observed in mild cases and low levels (0.7385pg/ml) in severe cases of co-infection of COVID-19 with parasites [23]. Gamma-interferon also plays a role in protective immunity against *Mycobacterium tuberculosis* and in potentially viral and anti-inflammatory activity [24]. Yamada *et al*. in 2020 found higher serum gamma-interferon concentration in patients who are likely to have a more severe local inflammatory response and elevated production in the bloodstream [25]. In the current study, the gamma-interferon level was normal (<0.58 μg/ml) in the serum of one of the majority of patients with pulmonary tuberculosis, unlike those co-infected with SARS-COV-2 who had a high gamma-interferon level (>0.58 μg/ml), thus corroborating the antiviral role of this cytokine.

The main comorbidity found in cases of COVID-19/TB co-infections were HIV/AIDS (26.7%), hypertension (20.3%), renal pathology (24.1%), diabetes mellitus (32.4 %), asthma (17.8%, P = 0.017), i.e. more than half of the patients presenting at least one comorbidity; a history of Bacille Calmette-Guérin (BCG) vaccination was present in 23.5% of the co-infected and a history of tuberculosis in 26.7%. Stochino *et al*. found comorbidity in 33.3% of co-infected. For 12.5% of the patients, it was a history of vaccination with Bacille Calmette-Guérin (BCG), but no case of co-infection COVID-19/tuberculosis/HIV was reported. Unlike Tadolini *et al*. in Belgium who found HIV/AIDS co-infection in 12.5% of patients approaching our finding. The other comorbidities were diabetes mellitus, asthma and a history of TB including 16.3%, 17.0% and 26.53% respectively[19]. All of these factors have been described in the literature as risk factors for TB [26]. Chen *et al*. in 2020 in China, reported comorbidities of 25% for diabetes mellitus, 22.2% for hypertension and 8.3% for past COVID-19 infection [27]. These disparities could be explained by the different socio-demographic contexts, indeed the distribution of chronic pathologies differs from one country to another, the same is true for vaccination policies against BCG [28].

During the COVID-19/TB co-infection, the most represented signs and symptoms were cough, asthenia and chest pain with respective prevalences of 22.6%, 29.5% and 26.1%. These symptoms were different from those found by Kunst *et al*. in 2020 in Belgium in a descriptive study in which the major symptoms were fever (81.2%), dry cough (56.2%) and dyspnea (15.7%) [19]. Various studies on the habits of use of antipyretics in Africa and particularly in Cameroon could justify this discrepancy, in fact, the use of these substances could temporarily mask fever during SARS-CoV-2 pathology [29].

Asthma and sore throat were statistically significant aggravating factors for SARS-CoV-2 carriage, and cough was a statistically significant protective factor for SARS-CoV-2 carriage in tuberculosis patients. In several previous studies, asthma does not appear to be a risk factor significantly associated with lung damage [30-32]. Pathophysiology could explain these differences with our findings. Asthma and sore throat are important anti-inflammatory components that induce the production of interferon which promotes the carriage of SARS-CoV-2. Cough thus prevents the installation of bacteria in the respiratory tract. The current study should be interpreted in the context of its limitations such as its cross-sectional design and the impossibility of either inferring causality or untangling the bi-directional relationship of risk factors for COVID-19/TB co-infection. Its strengths include robust methods using standard laboratory diagnostic tests (e.g. RT-PCR) for COVID-19 and logistic regression to prevent bias in our results.

## Conclusion

At the end of our work, which focused on the prevalence and factors associated with the carriage of SARS-CoV-2 in tuberculosis patients, it appears that the most represented age group in the Respiratory Diseases Center of Douala was 45 to 55 years and HIV/AIDS and hypertension were the comorbidities mainly represented in the population. The prevalence of SARS-CoV-2 RNA in pulmonary tuberculosis patients diagnosed was 24.3%, more than the national prevalence. In multivariate analysis, asthma, cough, and sore throat were significantly associated with SARS-CoV-2 RNA carriage. W, therefore, recommend a systematic screening of COVID-19 for all patients consulting in respiratory disease services and vaccination against COVID-19 for patients with TB after excluding an active infection.

**Limitations:** this being a cross-sectional study, the causal relationship between pulmonary infection and carriage of SARS-CoV-2 could not be deduced. The measurement of the interferon-gamma level by Interferon-Gamma Release Assays (IGRAs) method is more specific than a measurement by ELISA of the total level as done in our work. In order to limit selection biases, the questionnaire was completed by the principal investigator so as not to discourage the subjects who agreed to participate, and to encourage them to answer all the questions asked.

### What is known about this topic

- *Studies are still carrying up for a better knowledge of co-infected tuberculosis/COVID-19*.

### What this study adds

- *The co-infected TB/COVID-19 patient was very often on elderly HIV-positive male patient;*
- *Co-infection prevalence during the study period was high, and higher than the national prevalence;*
- *The associated aggravating factors were asthma and sore throat; cough was a protective factor for SARS-CoV-2 carriage in tuberculosis patients*.
